# Identification of oncogenes and tumor-suppressor genes with hepatocellular carcinoma: A comprehensive analysis based on TCGA and GEO datasets

**DOI:** 10.3389/fgene.2022.934883

**Published:** 2023-01-04

**Authors:** Yue Zhu, Yanfei Wang, Mengyao Hu, Xiaoting Lu, Guoping Sun

**Affiliations:** ^1^ Department of Oncology, The First Affiliated Hospital of Anhui Medical University, Hefei, Anhui, China; ^2^ Department of Integrated Traditional Chinese and Western Medicine, Anhui Medical University, Hefei, Anhui, China

**Keywords:** hepatocellular carcinoma, prognosis, diagnosis, PBK, F9

## Abstract

**Aim:** Existing targeted therapies for hepatocellular carcinoma (HCC) are resistant and have limitations. It is crucial to find new HCC-related target genes.

**Methods:** RNA-sequencing data of HCC were gathered from The Cancer Genome Atlas and Gene Expression Omnibus datasets. Initially, differentially expressed genes between normal and tumor tissues were identified from four Gene Expression Omnibus datasets, GSE36376, GSE102079, GSE54236, and GSE45267. GO terms and KEGG pathway enrichment analyses were performed to explore the potential biological functions of differentially expressed genes. A PPI network was constructed by using the STRING database, and up-regulated and down-regulated hub genes were defined through 12 topological approaches. Subsequently, the correlation bounded by up-regulated genes and down-regulated genes in the diagnosis, prognosis, and clinicopathological features of HCC was analyzed. Beyond a shadow of doubt, the key oncogene *PBK* and tumor suppressor gene *F9* were screened out, and the specific mechanism was investigated through GSEA enrichment analysis and immune correlation analysis. The role of *PBK* in HCC was further verified by western blot, CCK8, transwell, and tube formation experiments.

**Results:** *CDCA5*, *CDC20*, *PBK*, *PRC1*, *TOP2A*, and *NCAPG* are good indicators of HCC diagnosis and prognosis. The low expressions of *F9*, *AFM*, and *C8B* indicate malignant progression and poor prognosis of HCC. *PBK* was found to be closely related to *VEGF*, *VEGFR*, and *PDGFR* pathways. Experiments showed that *PBK* promotes HCC cell proliferation, migration, invasion, and tube formation in HUVEC cells. *F9* was negatively correlated with the degree of immune infiltration, and low expression of *F9* suggested a poor response to immunotherapy.

**Conclusion:** The role of HCC-related oncogenes and tumor-suppressor genes in diagnosis and prognosis was identified. In addition, we have found that *PBK* may promote tumor proliferation through angiogenesis and *F9* may be a predictor of tumor immunotherapy response.

## Introduction

Hepatocellular carcinoma (HCC) attracted widespread attention due to its high incidence rate and hovering mortality rate (Siegel et al., 2022). According to the global cancer statistics in 2020, the mortality of HCC ranks third, up to 8.3% (Sung et al., 2021). Although the first-line therapy of liver cancer continues to provide breakthroughs, its long-term survival is still unsatisfactory. Gene and molecular targeted therapy of HCC is the focus of current research. Advances in molecular targeted therapies grant us to further make explorations of precise molecular mechanisms of carcinogenesis and facilitate us to identify potential diagnostic and prognostic biomarkers for HCC (Llovet et al., 2018), which requires us to further reinforce the identification and screening of oncogenes and tumor-suppressor genes.

Oncogenes and tumor-suppressor genes act against each other, preserve balance, and accurately regulate the growth, proliferation, and apoptosis of normal cells. When mutations or changes in the expression of oncogenes or oncogenes thus disrupt this balance, cancers can be caused (Baeissa et al., 2016).

Currently, public data and bioinformatic analysis methods have provided us with invaluable resources to find HCC-related oncogenes and tumor-suppressor genes. Some studies searched cancer-related target genes through public databases, whether prognostic oncogene identification (Gao et al., 2021; Yan et al., 2021; Shen et al., 2022a; Gao et al., 2022), single-gene research studies (Ding et al., 2022; Yang et al., 2022), or immune-related oncogenes (Chen et al., 2020; Shen et al., 2022b; Long et al., 2022), all of which provided a strong basis for targeted therapy and precise treatment of liver cancer. However, many studies only consider the differential expression of oncogenes in tumors, ignoring the important role and potential association of tumor-suppressor genes. In addition, with continuous refreshing of the sequencing results and the gradual improvement of technology, the joint analysis of the combined multinomial databases makes the target gene prediction more accurate and reliable. Continuous exploration and updating of the prediction of HCC target genes is warranted.

In the present study, we identified DEGs based on four datasets from GEO: GSE36376, GSE102079, GSE54236, and GSE45267. Gene Ontology (GO) and Kyoto Encyclopedia of Genes and Genomes (KEGG) pathway enrichment analyses were performed subsequently. Afterward, we used the STRING database to construct a protein–protein interaction (PPI) network and identified six up-regulated and six down-regulated hub genes by using Cytoscape software; the correlation between these hub genes and clinical indexes such as clinical-stage, histological grade, as well as clinical status was investigated. Then, we built a prognostic model and screened prognostic genes by analyzing the overall survival (OS), disease-free survival (DSS), and progress-free interval (PFI) indicators. Convincingly, we identified six oncogenes and three tumor-suppressor genes as major prognostic biomarkers. Meanwhile, the mechanisms of key genes *PBK* and *F9* were also focused on and explored, and it was determined that *PBK* may promote the proliferation of HCC through angiogenesis. *F9* was associated with immunotherapy for HCC, and a low expression of *F9* represents lower reactivity to immune checkpoint inhibitors.

## Materials and methods

### Cell culture

MHCC97H cells were purchased from the Chinese Academy of Sciences (Shanghai, China). The HUVECs were from Bioogenetech (Shanghai, China). These cells were placed in an incubator at 37°C and 5% carbon dioxide.

### Small-interference RNA transfection

Small-interfering RNA targeting *PBK* was constructed from the RiboBio Co., Ltd. (Guangzhou, China). The cells were spread onto six-well plates and grown to 60%. Transfection was performed using Lipofectamine™ 2000 (Invitrogen, United States) according to the instructions. The siRNA target sequence is described in Supplementary Table S1.

### Cell viability analysis

Cells were seeded onto 96-well plates (3 × 10^3^ cells/well). The cell proliferation rate was determined by CCK8 solution (BestBio, Shanghai, China), and the absorbance was measured at 450 nm.

### Transwell assay

The MHCC97H cells were re-suspended in a medium with 5% FBS and placed in the upper chambers. The bottom chamber was filled with a medium with 20% FBS. After 24 h, the migrating cells were fixed with formaldehyde, stained with crystal violet, and counted. The invasion assay was performed in the same way, and the bottom of the chamber was covered with 1:6 dilution of Matrigel (Corning, United States).

### Tube formation assay

Medium supernatants from the control group and *PBK* knockdown group of MHCC97H cells were collected. The tube formation assay was performed according to the given method (Chen et al., 2021). The number of branches was analyzed using WimTube (https://www.wimasis.com).

### Western blot

A western blot analysis was performed (Zhu et al., 2020). Antibodies were all purchased from Proteintech (Wuhan, China).

### Data acquisition and processing

The NCBI GEO database (http://www.ncbi.nlm.nih.gov/geo) is an open-access platform for data. RNA-sequencing datasets containing HCC tissue samples and normal tissue samples were obtained from the GEO database, including GSE36376, GSE102079, GSE54236, and GSE45267, and were downloaded for further analysis. DEG mRNA expressions were detected in 371 HCC tissues and 50 normal liver tissues from The Cancer Genome Atlas (TCGA) database.

### Identification of differentially expressed genes

The limma package of R software was used to identify the DEGs in four datasets. Adj *p* value <..05 and | log2 fold change (FC) | ≥ 1 were set as the threshold values of DEG identification. Then, OmicStudio tools (https://www.omicstudio.cn/tool) were used to make Venn diagrams. Four dataset-intersection genes were extracted.

### Enrichment analysis of differentially expressed genes

The clusterProfiler package (version 3.14.3) of R software was used for the enrichment analysis with the following ontology sources: GO biological processes (BPs), cellular components (CCs), molecular functions (MFs), and KEGG pathway. Using *p* < .05 and count ≥2 as cut-off values, we identified the DEG-enriched pathways.

### Pathway activation level calculation

Pathway activation level (PAL) is a metric allowing the direct calculation of pathway activity levels using high-throughput gene expression data. A positive value for PAL indicates activation of the pathway, while a negative value indicates inhibition of the pathway. PAL values were obtained based on the OncoboxPD (https://open.oncobox.com) for analysis of KEGG enrichment pathways (Zolotovskaia et al., 2022).

### Protein–protein interaction network and hub genes

PPI networks of up-regulated genes and down-regulated genes were generated using the STRING database. The hub gene was defined by 12 topological methods (MCC, DMNC, MNC, Degree, EPC, BottleNeck, EcCentricity, Closeness, Radiality, Betweeness, Stress, and Clustering) using Cytoscape software.

### Clinicopathological features and prognosis analysis

RNA-seq and clinical data of 424 cases of liver cancer from the TCGA database (https://portal.gdc.cancer.gov/) were analyzed. We conducted the clinicopathological features and survival analysis, including the T stage, pathologic stage, histological grade, AFP, vascular invasion, tumor statues, prothrombin time (PT), race, OS, DSS, and PFI, with the Xiantao Academic (https://www.xiantao.love/) platform (survminer package of R software).

### Gene correlation analysis

RNA-sequencing expression profiles and the corresponding clinical information for liver cancer were downloaded from the TCGA dataset. The multi-gene correlation pheatmap is displayed by using the R software package. *p* values less than .05 were considered statistically significant.

### Correlation analysis of genes and pathways

RNA-sequencing expression profiles and the corresponding clinical information for liver cancer were downloaded from the TCGA dataset. R software GSVA package was used to analyze, choosing parameter as method = “ssgsea.” All the analytic methods and R packages were implemented by R version 4.0.3.

### ICB response

The Tumor Immune Dysfunction and Exclusion (TIDE) algorithm was used to predict the response of individual samples or groups to immune checkpoint inhibitors based on expression profile data (Jiang et al., 2018). R (V4.0.3) software packages GGploT2 (v3.3.3) and GGPUBr (0.4.0) were used for mapping analysis.

### Statistical analysis

The two-tailed independent-sample t-test or Wilcoxon rank sum test was used to compare the mean difference between the two groups. The association between genes and clinicopathological parameters was evaluated using the Pearson Chi-square test or Fisher exact test. Spearman correlation analysis was used in different expressions of genes and pathways. Kaplan–Meier survival analysis was made by using log-rank test. The major analysis was performed using Xiantao Academic (https://www.xiantao.love/) in this study. Differences were considered statistically significant at *p < .05, **p < .01, and ***p < .001.

## Results

### Identification of DEGs

**FIGURE 1:**
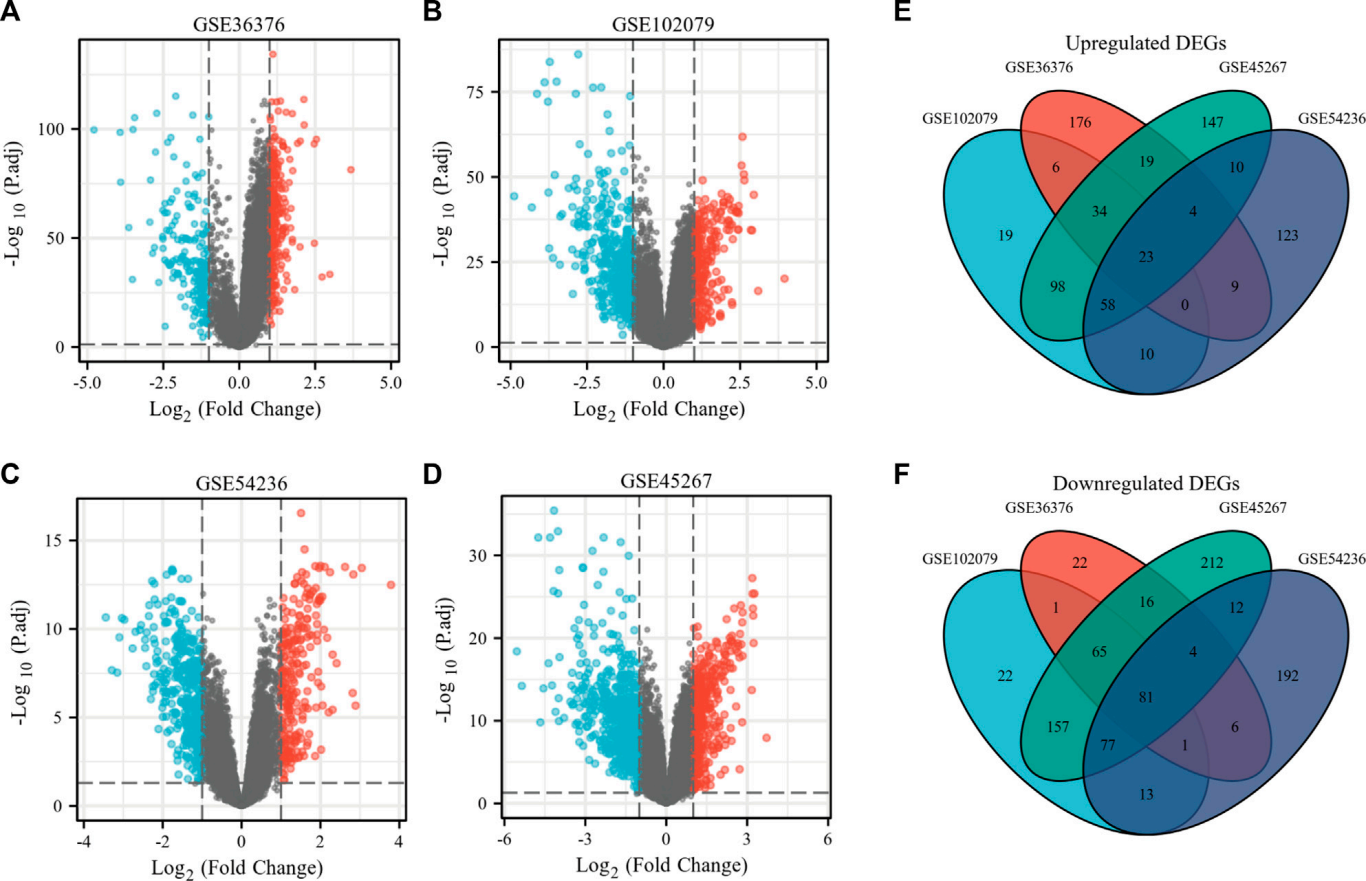
Identification of overlapping DEGs in HCC. **(A–D)** Volcano plots of gene expression profile data in GSE36376 **(A)**, GSE102079 **(B)**, GSE54236 **(C)**, and GSE45267 **(D)**, respectively. **(E)** Venn plots of up-regulated overlapping DEGs. **(F)** Venn plots of down-regulated overlapping DEGs.

To make a comparison between the difference in gene expression between the HCC group and the normal group, differential gene expression analysis within samples was executed. The present study involved four GEO datasets, GSE36376, GSE102079, GSE54236, and GSE45267. GSE36376 contained 240 HCC tumor tissue samples and 193 adjacent non-tumor tissues. GSE102079 contained 152 HCC tumor tissue samples, 91 adjacent non-tumor tissues, and 14 normal liver tissues. Furthermore, GSE54236 contained 81 HCC tumor tissue samples and 80 normal liver tissue samples. Furthermore, GSE45267 contained 48 HCC tumor tissues and 39 normal liver tissue samples. Thereby, we counted the differential genes and collected the data to prove that 271 genes were up-regulated and 196 genes were down-regulated in the GSE36376 database (Figure 1A). In the GSE102079 database, 248 genes were up-regulated and 417 genes were down-regulated (Figure 1B). In the GSE54236 (Figure 1C) and GSE45267 databases (Figure 1D), 237 and 393 genes were up-regulated and 386 and 624 genes were down-regulated, respectively. As presented in Figures 1E, F, we made an attempt to identify 104 DEGs, including 23 up-regulated genes and 81 down-regulated genes in the HCC tissue samples.

### GO and KEGG pathway enrichment analyses of DEGs

**FIGURE 2:**
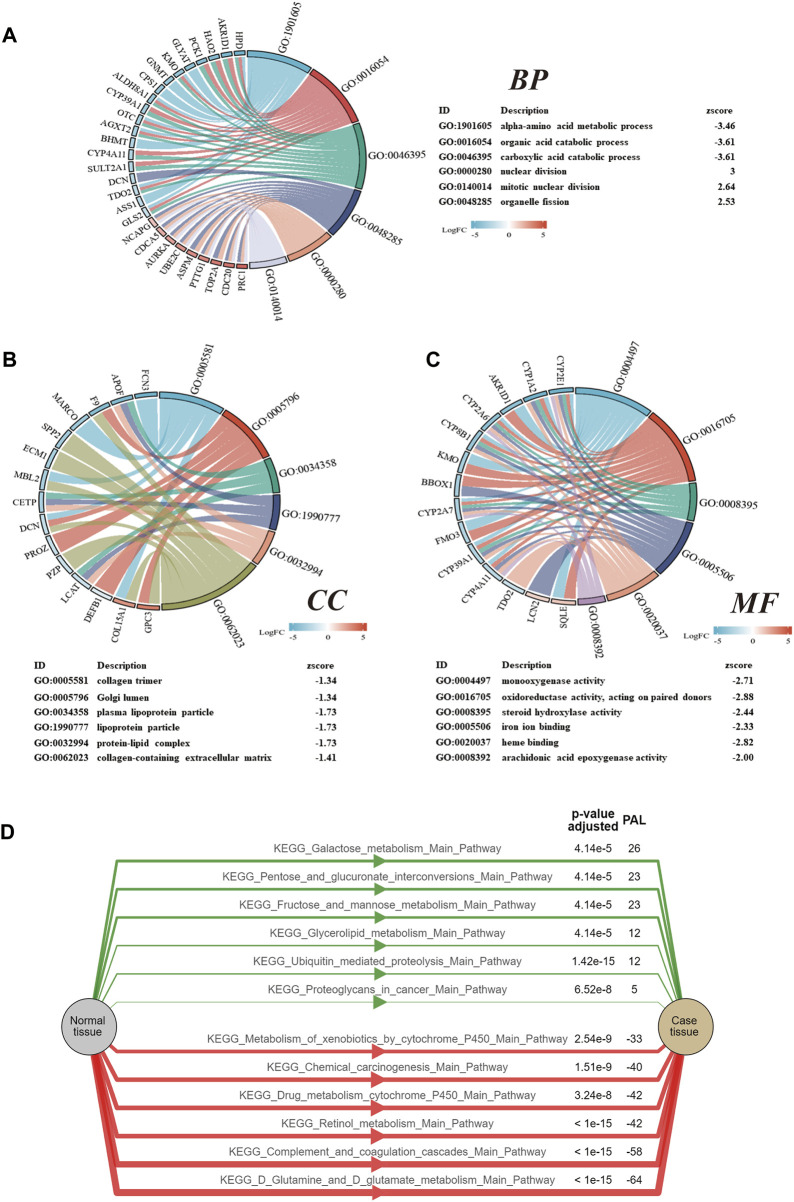
GO and KEGG analyses of the DEGs. **(A)** Chord diagrams of the biological process. **(B)** Chord diagrams of the cellular components. **(C)** Chord diagrams of the molecular function. **(D)** KEGG pathway analysis of the DEGs. A positive value for PAL indicates activation of the pathway, while a negative value indicates inhibition of the pathway.

In order to further study the potential biological functions of the DEGs, GO terms and KEGG pathway enrichment analyses were settled. The enriched GO and enriched KEGG functions in the ClusterProfiler package were adapted to enrich DEGs from BP, CC, MF, and KEGG. According to the results, they revealed that the biological processes primarily associated with the DEGs were alpha-amino acid metabolic, organic acid catabolic, and carboxylic acid catabolic (Figure 2A). These DEGs have also been closely associated with the cellular components of collagen trimer, Golgi lumen, and plasma lipoprotein particle (Figure 2B). The top molecular functions involved include monooxygenase activity, oxidoreductase activity, and steroid hydroxylase activity (Figure 2C). At the same time, Chord diagrams were used to display the genes enriched in the top six processes of BPs, CCs, and MFs. PAL values were used to analyze the KEGG enrichment pathway; PAL values were also used to analyze the KEGG enrichment pathway. The KEGG pathway analysis proved the DEGs were significantly enriched in glutamate metabolism, complement and coagulation cascades, and retinol metabolism (Figure 2D).

### The construction of the PPI network and the identification of hub genes

**FIGURE 3:**
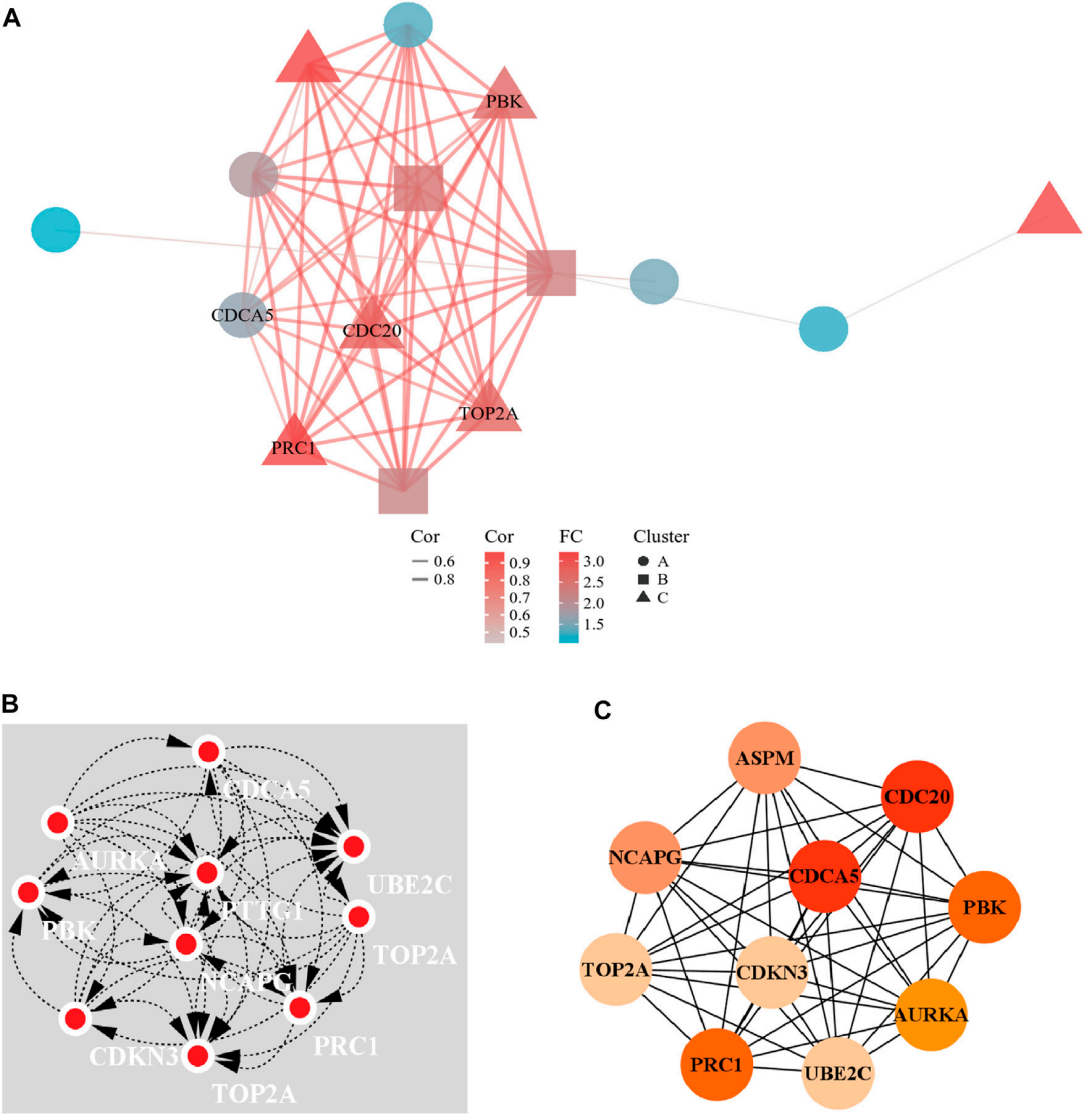
The PPI network of up-regulated DEGs and the interaction network of top 10 hub genes. **(A)** The PPI network of the 23 up-regulated DEGs. **(B)** Top 10 hub gene network constructed by clustering topological method. **(C)** Top 10 hub gene network constructed by EPC topological method.

**FIGURE 4:**
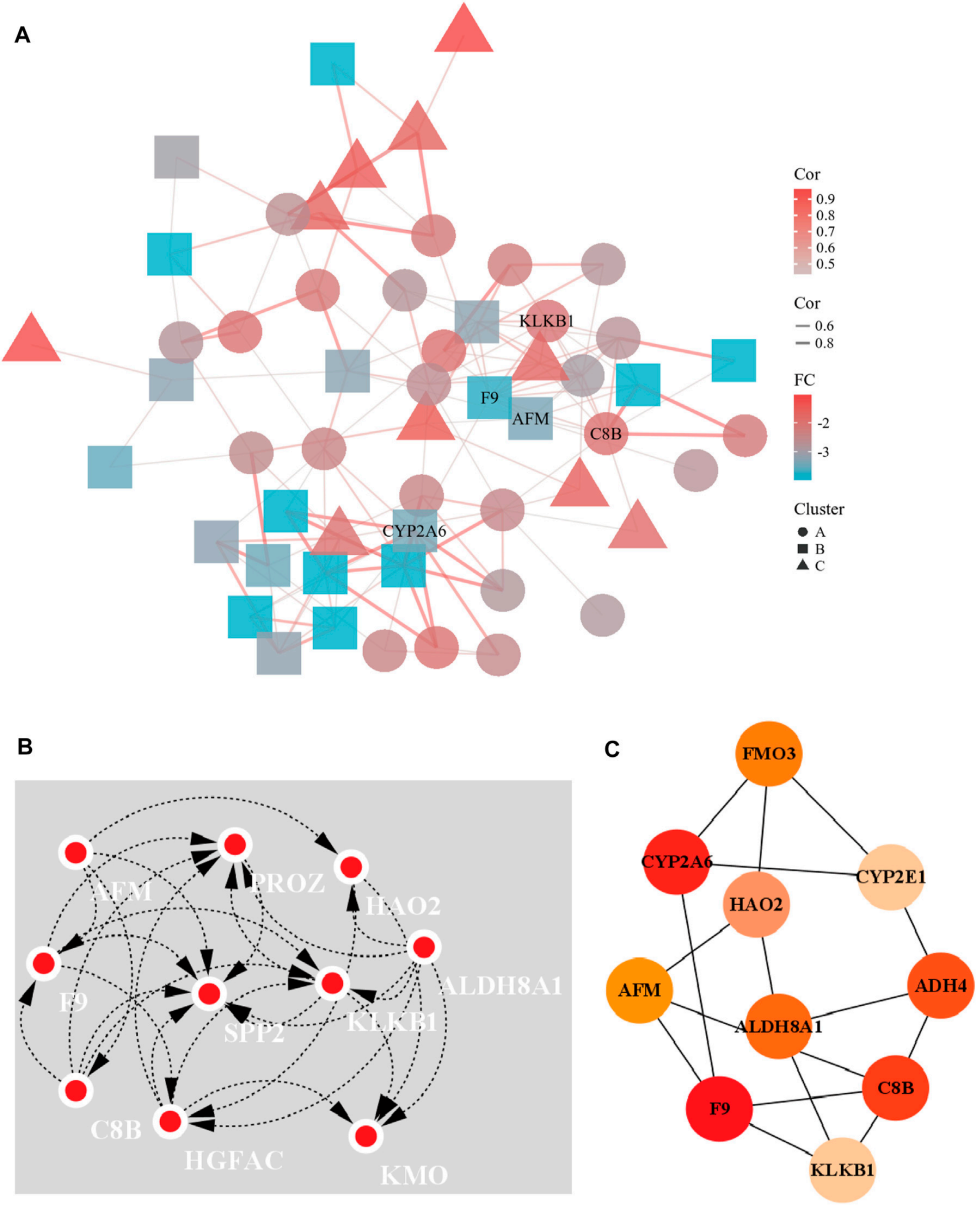
The PPI network of down-regulated DEGs and the interaction network of hub genes. **(A)** The PPI network of the 81 down-regulated DEGs. **(B)** Eccentricity topological method was used to identify down-regulated hub genes. **(C)** Top 10 hub gene network constructed by closeness topological method.

A PPI network of 23 up-regulated genes was constructed by adapting the STRING database. The PPI network consists of 15 nodes and 59 edges (Figure 3A), and an interaction score >0.7 was regarded as a high-confidence interaction relationship. Hub genes were measured using 12 topological methods of the CytoHubba plug-in in Cytoscape (Table 1). As presented in Figures 3B, C, there are six genes identified as up-regulated hub genes, namely, *PBK*, *PRC1*, *CDCA5*, *CDC20*, *TOP2A*, and *NCAPG*. Then, a PPI network of 81 down-regulated genes, containing a total of 54 nodes and 154 edges, was generated by applying the STRING databas(Figure 4A). The 12 topological methods were employed to screen hub genes (Table 2). Six genes with the highest frequencies were selected as down-regulated hub genes, namely, *KLKB1*, *F9*, *ALDH8A1*, *C8B*, *AFM*, and *CYP2A6* (Figures 4B, C).

**TABLE 1:** Top 10 up-regulated DEGs by using 12 topological analysis methods of CytoHubba.

MCC	DMNC	MNC	Degree	EPC	BottleNeck	Eccentricity	Closeness	Radiality	Betweeness	Stress	Clustering
PTTG1	PTTG1	PTTG1	PTTG1	CDCA5	CDCA5	CDCA5	CDCA5	CDCA5	CDCA5	CDCA5	PTTG1
PBK	PBK	PBK	PBK	CDC20	CDC20	CDC20	CDC20	CDC20	CDC20	CDC20	PBK
PRC1	PRC1	PRC1	PRC1	PBK	PTTG1	TOP2A	TOP2A	TOP2A	TOP2A	TOP2A	NCAPG
CDCA5	CDCA5	CDCA5	CDCA5	PRC1	PBK	PTTG1	NCAPG	NCAPG	NCAPG	NCAPG	PRC1
CDC20	CDC20	CDC20	CDC20	NCAPG	PRC1	PBK	PTTG1	PTTG1	PTTG1	PTTG1	CDC20
ASPM	ASPM	ASPM	ASPM	TOP2A	NCAPG	PRC1	PBK	PBK	PBK	PBK	CDCA5
AURKA	AURKA	AURKA	AURKA	ASPM	TOP2A	ASPM	PRC1	PRC1	PRC1	PRC1	TOP2A
TOP2A	TOP2A	TOP2A	TOP2A	AURKA	ASPM	NCAPG	ASPM	ASPM	ASPM	ASPM	AURKA
CDKN3	CDKN3	CDKN3	CDKN3	CDKN3	IGF2BP3	AURKA	AURKA	AURKA	IGF2BP3	IGF2BP3	CDKN3
NCAPG	NCAPG	NCAPG	NCAPG	UBE2C	CCL20	IGF2BP3	CDKN3	CDKN3	CCL20	CCL20	UBE2C

**TABLE 2:** Top 10 down-regulated DEGs by using 12 topological analysis methods of CytoHubba.

MCC	DMNC	MNC	Degree	EPC	BottleNeck	Eccentricity	Closeness	Radiality	Betweeness	Stress	Clustering
SPP2	PROZ	CYP2A6	C8B	F9	F9	KLKB1	F9	F9	F9	F9	MT1F
PROZ	F9	CYP2E1	CYP2A6	ALDH8A1	FMO3	HGFAC	CYP2A6	HAO2	CYP2A6	CYP2A6	SLC38A4
MBL2	AFM	KLKB1	KLKB1	KLKB1	HAO2	ALDH8A1	C8B	ALDH8A1	FMO3	FMO3	APOF
KLKB1	SPP2	C8B	SPP2	SPP2	OTC	PROZ	ADH4	FMO3	ADH4	ADH4	CETP
HGFAC	NAT2	CYP1A2	F9	PROZ	CYP2A6	C8B	ALDH8A1	ADH4	HAO2	HAO2	NAT2
F9	FMO3	MBL2	CYP2E1	C8B	SRD5A2	KMO	FM03	CYP2A6	OTC	OTC	LCAT
CYP1A2	KLKB1	HGFAC	ALDH8A1	HGFAC	C8B	AFM	AFM	C8B	ALDH8A1	ALDH8A1	CNDP1
C9	MBL2	SPP2	CYP1A2	MBL2	BHMT	SPP2	HAO2	AFM	AFM	AFM	C7
C8B	HGFAC	F9	AFM	CYP2E1	CYP1A2	F9	CYP2E1	KLKB1	C8B	C8B	FCN3
AFM	C9	ALDH8A1	MBL2	AFM	CPS1	HAO2	KLKB1	HGFAC	AGXT2	AGXT2	MT1E

### Correlation between up-regulated genes and clinicopathological features and prognostic analysis

**FIGURE 5:**
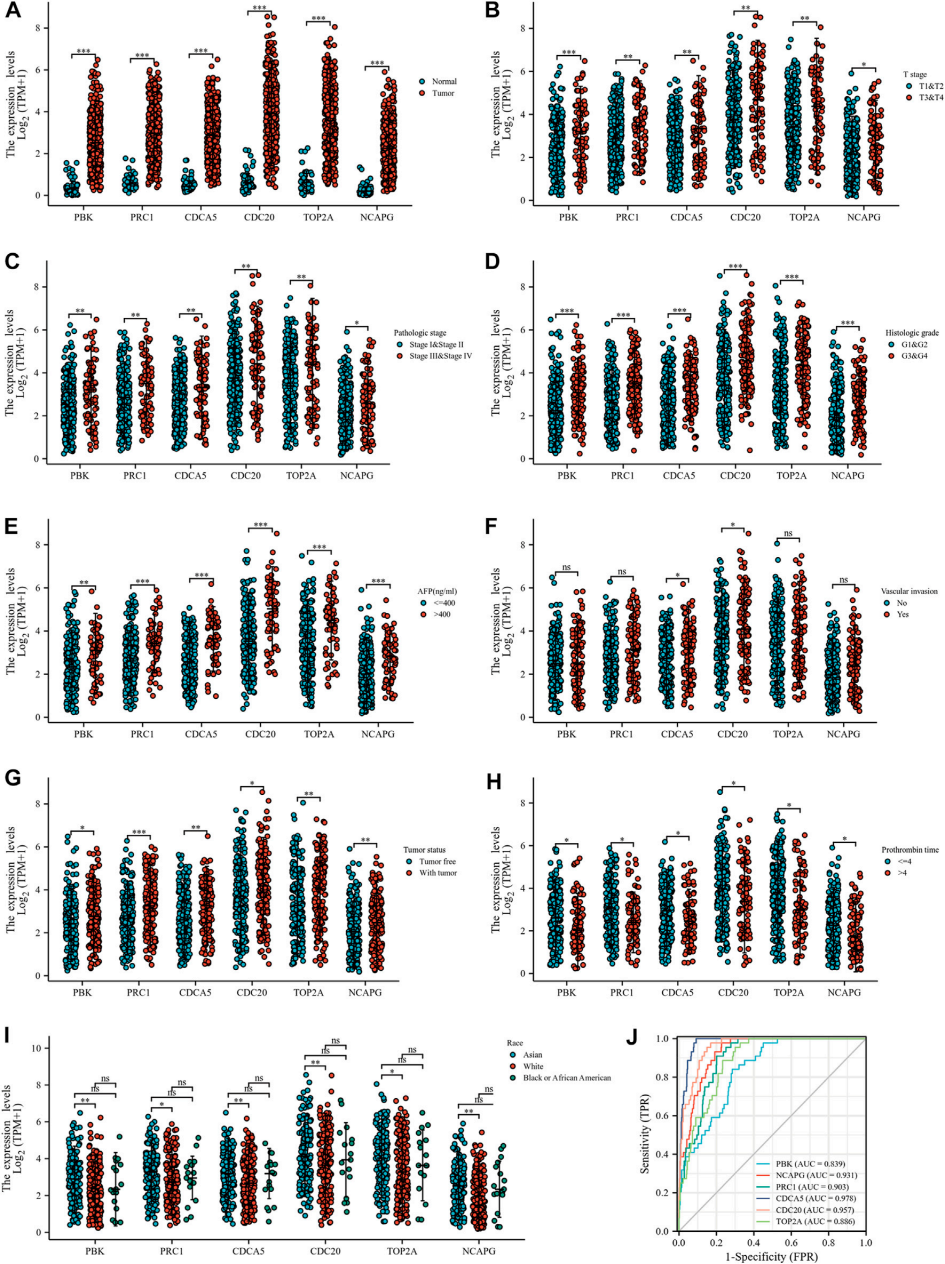
Correlation between up-regulated hub genes and clinicopathological features. **(A)** DEG expressions in HCC and normal tissues based on TCGA database. **(B–I)** Correlation between up-regulated DEGs and T stage **(B)**, pathologic stage **(C)**, histological grade **(D)**, AFP **(E)**, vascular invasion **(F)**, tumor status **(G)**, PT **(H)**, and race **(I)**. **(J)** ROC curve of up-regulated DEGs in the diagnosis of HCC.

**FIGURE 6:**
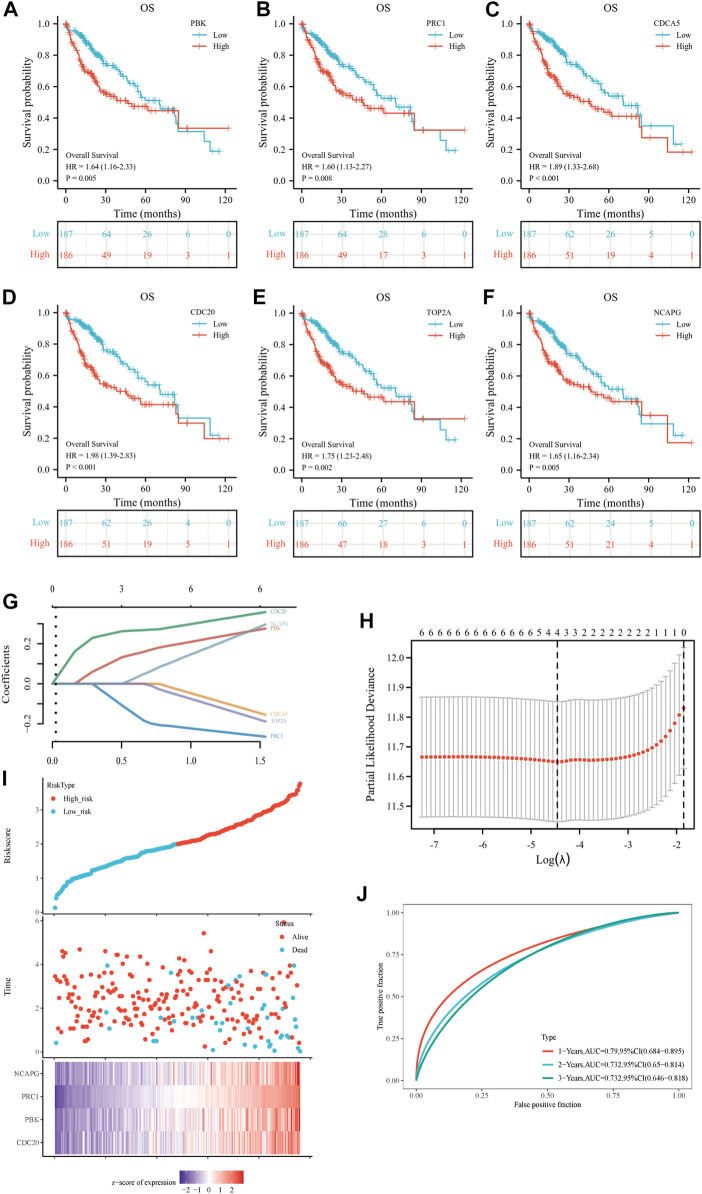
Prognostic gene signature of six up-regulated core genes in HCC patients. **(A–F)** Association between *PBK* **(A)**, *PRC1* **(B)**, *CDCA5* **(C)**, *CDC20* **(D)**, *TOP2A* **(E)**, and *NCAPG* **(F)** expression and overall survival (OS) were performed with the online Kaplan–Meier survival analysis (KMplot, http://kmplot.com/analysis/). **(G)** The calculation of the regression coefficient. **(H)** LASSO Cox regression model of the six hub genes. **(I)** The risk score, survival time, and survival status of the selected dataset. The top scatterplot represents the risk score from low to high. The scatter plot distribution represents the risk score of different samples corresponding to the survival time and survival status. The button heatmap is the gene expression from the signature. **(J)** The ROC curve and AUC of the model. The higher values of AUC correspond to higher predictive power.

The investigators evaluated the mRNA expression of six up-regulated genes in HCC tissue or normal liver tissue from all patient characteristics of TCGA (Figure 5A). We analyzed the correlation between up-regulated DEGs and the T stage (Figure 5B), pathologic stage (Figure 5C), histological grade (Figure 5D), AFP (Figure 5E), vascular invasion (Figure 5F), tumor status (Figure 5G), PT (Figure 5H), and race (Figure 5I). Six DEGs were highly expressed in HCC tissues and were significantly associated with the T stage, pathological stage, histological grade, AFP, tumor status, and PT. Except for those, the expressions of these genes were all above in Asians compared to Whites and were not statistically different in Blacks and African America. In addition, *CDCA5* and *CDC20* have a close relationship with the degree of vascular invasion. Hence, we further evaluated the specificity and sensitivity of the six genes in the diagnosis of their AUC values from high to low, namely: *CDCA5* (AUC = 0.978), *CDC20* (AUC = 0.957), *NCAPG* (AUC = 0.931), *PRC1* (AUC = 0.903), *TOP2A* (AUC = 0.886), and *PBK* (AUC = 0.839), implying that they all had acceptable specificity and sensitivity as diagnostic markers for HCC (Figure 5J). Furthermore, we conducted a Kaplan–Meier survival analysis with the candidate genes, and OS, DSS, and PFI were selected as prognostic indicators. As a consequence of the analysis, *PBK*, *PRC1*, CDC5, *CDC20*, *TOP2A*, and *NCAPG* were significantly correlated with OS (Figures 6A–F), DSS (Supplementary Figures S1A–F), and PFI (Supplementary Figures S1G–L), indicating that they were good predictors of the prognosis of HCC. Therefore, we constructed a prognostic model based on these six genes using the prognostic information of 240 HCC patients from the International Cancer Genome Consortium (ICGC) database. The calculation of the regression coefficient is depicted in Figure 6G. LASSO Cox regression model showed that this prognostic model had the best predictive ability when containing four genes (Figure 6H). A heatmap of gene expressions and the risk-score scatter plot indicate that high-gene expression was a high-risk group, whose corresponding patients had shorter survival time and more dead patients in clinical statuses (Figure 6I). It is worth noting that the ROC curve and AUC values also reflected that the prognostic model has strong predictive power for 1-year survival (AUC = 0.79), 2-year survival (AUC = 0.732), and 3-year survival (AUC = 0.732), with the best-fitted prediction power for 1-year survival (Figure 6J).

### The promoting effect of *PBK* in hepatocellular carcinoma may be related to angiogenesis

**FIGURE 7:**
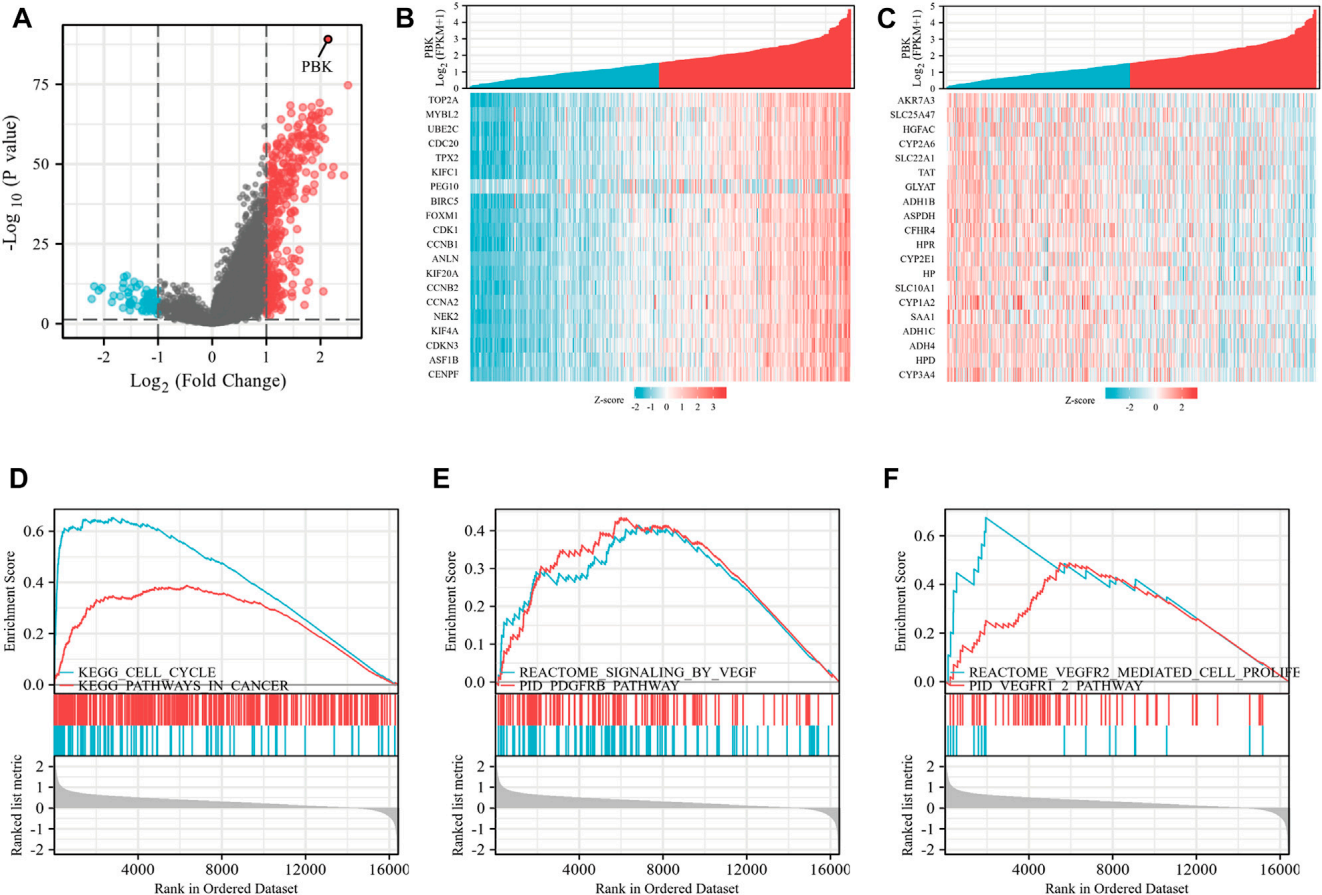
*PBK*-related differential gene and pathway enrichment. **(A)** Volcano maps were made according to the *PBK*-related differential genes. **(B)** Heatmap of the top 20 up-regulated genes. **(C)** The top 20 up-regulated genes were screened. **(D–F)** GSEA analysis based on the DEGs.

**FIGURE 8:**
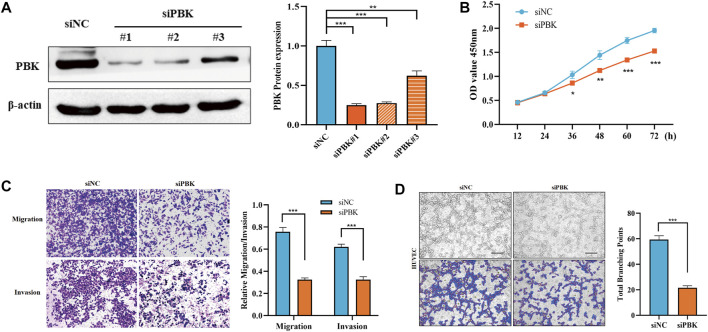
*PBK* promotes the malignant progression of HCC cells. **(A)** Western blot was used to detect the protein expression of *PBK* in the control and *PBK* knockdown groups. **(B)** CCK8 assay was used to detect the proliferation ability of HCC cells. **(C)** Transwell assay was used to detect the migration and invasion abilities of HCC cells. **(D)** Tube formation assay was performed in HUVEC cells.

Based on the expression of the genes in HCC and the establishment of the prognosis model, we selected *PBK* for further study in HCC. As shown in Figure 7A, differential genes of *PBK* high/low-expression groups were extracted from TCGA database, and volcano maps were made according to the FC values. The top 20 up-regulated and down-regulated genes were screened to make a correlation heatmap (Figures 7B, C). Subsequently, the gene set enrichment analysis (GSEA) was used to explore the main enrichment pathways of *PBK*. We found that *PBK* was enriched in the “signaling of *VEGF*,” “*PDGFRB*,” “*VEGFR* pathway” and “*VEGFR2*-mediated cell proliferation” pathways in addition to a significant correlation with “cell cycle” (Figures 7D–F). Therefore, we hypothesized that *PBK* may be related to angiogenesis and promote tumor proliferation through *VEGFR*-mediated vascular formation. In order to further verify the role of *PBK* in HCC, we used small-interfering RNA to knock down *PBK* in MHCC97H cells (Figure 8A). CCK8 assay showed that knocked down *PBK* could significantly inhibit the proliferation of HCC cells (Figure 8B). Transwell assay further proved that the invasion and migration ability of HCC cells were inhibited to varying degrees after *PBK* silencing (Figure 8C). Then, we collected the cell culture medium for tube formation assay, the HUVEC tube formation was decreased by adding the prepared *PB*K-knockdown MHCC97H cell supernatants, and this led to much fewer total branching points as quantified by the WimTube (Figure 8D), suggesting that *PBK* may be involved in tumor vasculogenesis.

### Correlation between down-regulated genes, clinicopathological features, and prognostic analysis

**FIGURE 9:**
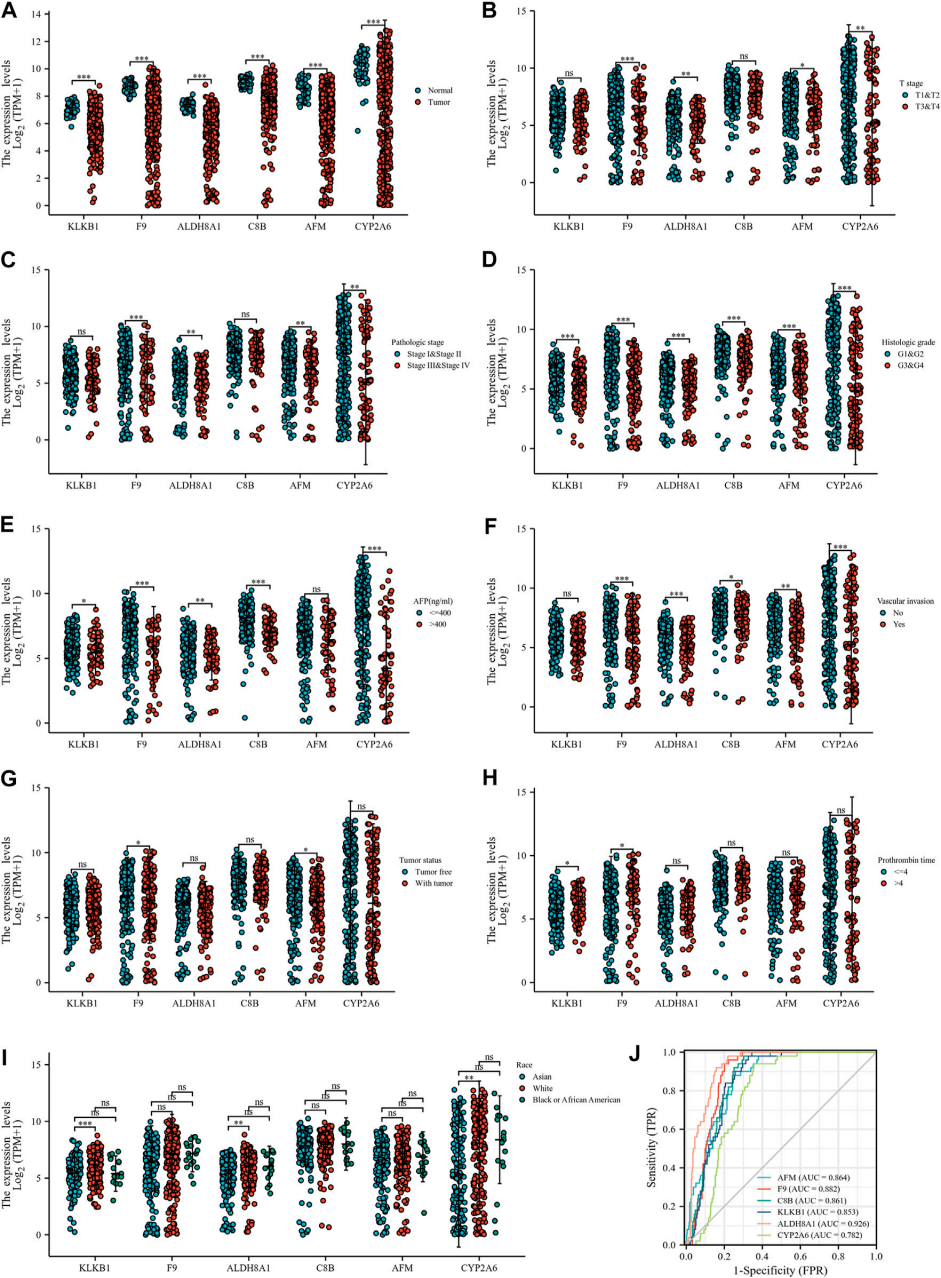
Correlation between down-regulated hub genes and clinicopathological features. **(A)** Down-regulated hub DEG expressions in HCC and normal tissues based on TCGA database. **(B–I)** Correlation between down-regulated hub genes and T stage **(B)**, pathologic stage **(C)**, histological grade **(D)**, AFP **(E)**, vascular invasion **(F)**, tumor status **(G)**, PT **(H)**, and race **(I)**. **(J)** ROC curve of down-regulated DEGs in the diagnosis of HCC.

**FIGURE 10:**
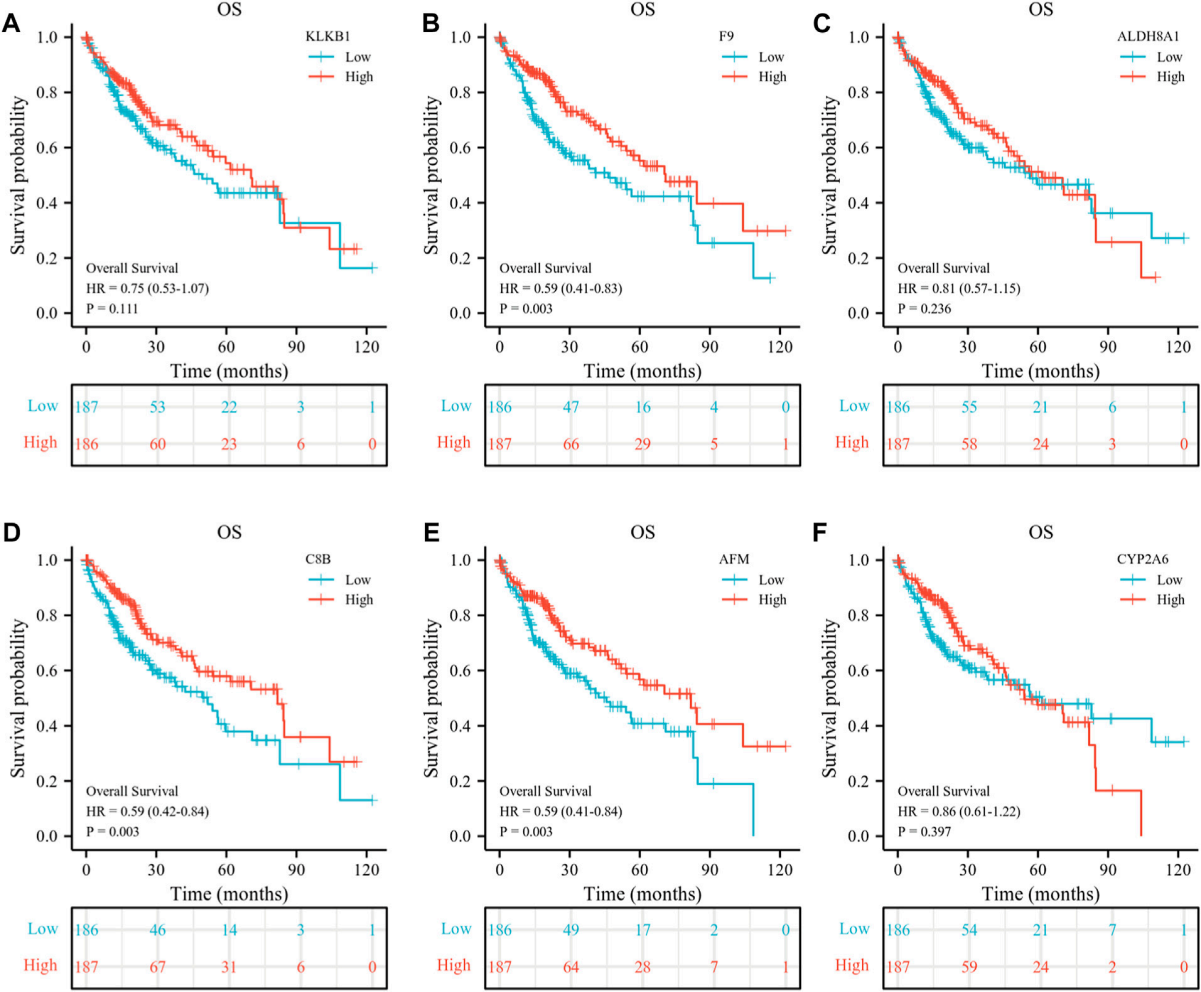
Prognostic analysis of six down-regulated hub genes. **(A–F)** Association between *KLKB1* **(A)**, *F9* **(B)**, *ALDH8A1* **(C)**, *C8B* **(D)**, *AFM* **(E)**, and *CYP2A6* **(F)** expressions and overall survival (OS) were performed with the Kaplan–Meier survival analysis.

At the same time, we detected the expression of six down-regulated hub genes in HCC and normal liver tissues by the TCGA database, the results suggested that *KLKB1*, *F9*, *ALDH8A1*, *C8B*, *AFM*, and *CYP2A6* were all more highly expressed in normal liver tissues than in cancerous tissues (Figure 9A). To further confirm the conjecture, we managed to find the correlation between down-regulated DEGs and T stage (Figure 9B), pathologic stage (Figure 9C), histological grade (Figure 9D), AFP (Figure 9E), vascular invasion (Figure 9F), tumor status (Figure 9G), PT (Figure 9H), and race (Figure 9I). The results showed that the six genes were closely related to histological grade. *F9*, *ALDH8A1*, *AFM*, and *CYP2A6* were statistically significant with T stage and pathologic stage. *KLKB1*, *F9*, *ALDH8A1*, *C8B*, and *CYP2A6* were significantly correlated with AFP. All genes were negatively associated with vascular invasion, except *KLKB1*. *F9* and *AFM* were closely related to tumor status, and *KLKB1* and *F9* were associated with PT. Furthermore, *KLKB1*, *ALDH8A1*, and *CYP2A6* were differentially expressed between Asians and Whites, and there was no racial variance in the expression of other genes. ROC curves were constructed and AUC values from high to low were: *ALDH8A1* (AUC = 0.926), *F9* (AUC = 0.882), *AFM* (AUC = 0.864), *C8B* (AUC = 0.861), *KLKB1* (AUC = 0.853), and *CYP2A6* (AUC = 0.782); AUC values larger than 0.7 were reckoned to have high diagnostic sensitivity and specificity, and all genes met the aforementioned requirements (Figure 9J). Moreover, we then performed a Kaplan–Meier survival analysis (Figures 10A–F). *F9*, *C8B*, and *AFM* were found to be correlated with prognostic indicators OS, DSS (Supplementary Figures S2A–C), and PFI (Supplementary Figures S2D–F), while *KLKB1*, *ALDH8A1*, and *CYP2A6* were not statistically significant (Supplementary Figures S2G–L).

### The relationship between immune cell infiltration and *F9* expression

**FIGURE 11:**
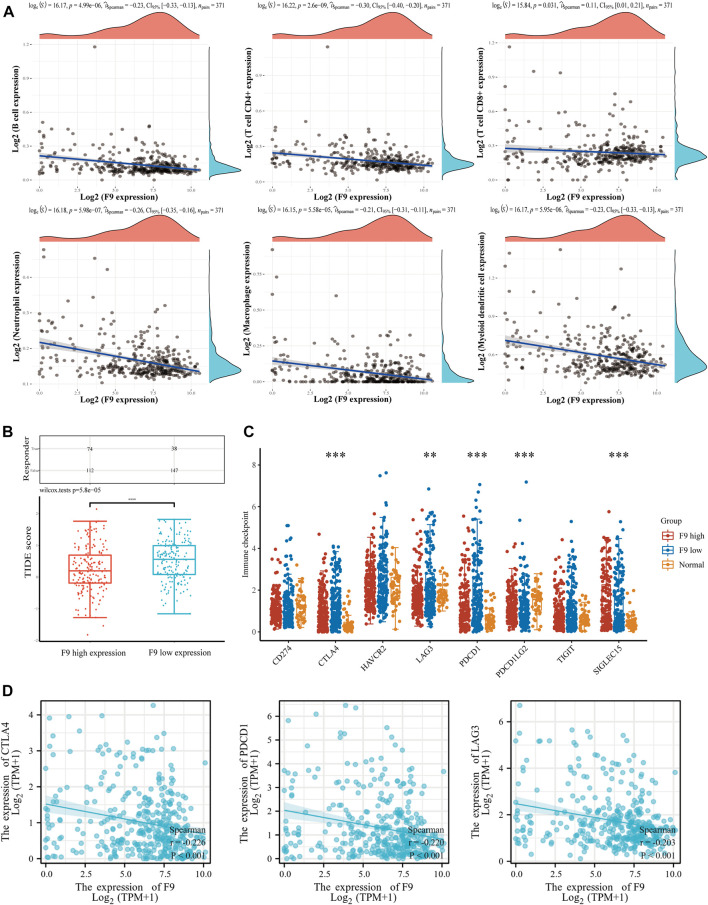
The correlation analysis between *F9* and immune cell infiltration in HCC. **(A)** The correlation between *F9* and immunocyte infiltration. **(B)** The distribution of immune response scores in *F9*-high and *F9*-low groups in the prediction results. The *F9*-low group was significantly higher than the *F9*-high-expression group. **(C)** The expression distribution of immune checkpoints gene in *F9*-high and *F9*-low tissues. **(D)** The correlation between *CTLA4*, *PD1*, *LAG3* and *F9* in HCC.

Scores of immune cell infiltration in HCC patients were investigated from the TCGA database. The expression of *F9* was negatively correlated with the infiltration level of CD4^+^ T cells (cor = −0.30, *p* = 2.60e-09), neutrophils (cor = −0.26, *p* = 5.98e-07), B cells (cor = −0.23, *p* = 4.99e-06), dendritic cells (cor = −0.23, *p* = 5.95e-06), macrophages (cor = −0.21, *p* = 5.58e-05), and CD8^+^ T cells (cor = −0.11, *p* = 3.1e-02) in HCC tissues (Figure 11A). Furthermore, the TIDE scores were higher in the *F9*-low-expression group contrasted with the *F9*-high-expression group, indicating that the *F9*-low-expression group had a worse response to immune checkpoint blocking therapy (ICB) (Figure 11B). Further study studies displayed that immune checkpoint-related genes *PD-1*, *CTLA4*, and *LAG3* were lower in the *F9*-high-expression group (Figure 11C), and were negatively correlated with *F9* in HCC by correlation analysis (Figure 11D).

## Discussion

HCC is one of the most malignant tumors. In recent years, the treatment of unresectable HCC mainly focuses on molecular targeted therapy (Huang et al., 2020; Zhao et al., 2020), such as lenvatinib and sorafenib, however, targeted drug resistance still exists (Jin et al., 2021). There is an urgent need to find new targets for the diagnosis and treatment of liver cancer in the direction of a breakthrough in the current treatment limitations. Some previous studies used TCGA and GEO databases to screen oncogenes and identify prognostic or immune-related genes (Chen et al., 2020; Gao et al., 2021; Yan et al., 2021; Shen et al., 2022a; Shen et al., 2022b; Ding et al., 2022; Gao et al., 2022; Long et al., 2022; Yang et al., 2022). However, in this study, four GSE datasets, GSE36376, GSE102079, GSE54236, and GSE45267, were summarized for analysis, hoping to find the mechanism of cancer-promoting and tumor-suppressor factors in liver cancer from the perspective of improving the accuracy of the research results. Here, we summarize the differences between this project and previous research studies (Supplementary Table S2).

The study yielded 23 up-regulated DEGs and 81 down-regulated DEGs. Subsequently, we defined six up-regulated hub genes *PBK*, *PRC1*, *CDC20*, *CDCA5*, *TOP2A*, and *NCAPG* and six down-regulated hub genes *KLKB1*, *F9*, *AFM*, *C8B*, *ALDH8A1*, and *CYP2A6* as the main subjects through 12 topological approaches. According to the study, the six up-regulated hub genes were significantly correlated with T stage, pathologic stage, histological grade, AFP, tumor status, and PT. *CDCA5* and *CDC20* were also associated with the degree of vascular invasion. The prognostic model based on these six genes showed results that the pivotal genes were closely associated with the survival time of hepatocellular carcinoma, especially for a short-term survival time, where the AUC value of the prognostic model was 0.79, showing a strong predictive power. In the ROC curve, the AUC values were all greater than 0.7, indicating that these six genes are good diagnostic and prognostic factors and are related to the malignant progression of HCC. Principally, *CDCA5* (AUC = 0.978) and *CDC20* (AUC = 0.957) showed excellent diagnostic specificity and sensitivity and could be considered key oncogenic genes in liver cancer.

Cell division cycle 20 homologous (*CDC20*), an E3 ring finger ubiquitin ligase, is one of the most significant factors controlling the spindle assembly checkpoint (SAC) (Hwang et al., 1998). *CDC20* overexpression stops mitosis and prevents apoptosis in DNA-damaged cells (Connors et al., 2014). Moreover, studies have made clear that *CDC20* is highly expressed in HCC and regulates the proliferation of P53-mutated HCC cells through the Bcl-2/Bax pathway (Zhao et al., 2021). Shi et al. (2021) have found that *CDC20* stabilizes *HIF-1A* protein under normoxic conditions in liver cancer tissues, and depletion of *CDC20* leads to tumor growth retardation and inactivation of the *HIF-1A* signaling pathway. These studies indicate that *CDC20* is a key role in the link of the malignant progression of liver cancer, which is consistent with our previous prediction.

Cell division cycle associated 5 (*CDCA5*) is a core regulator of DNA repair and chromosome separation, and plays a carcinogenic role in gastric cancer (Zhang et al., 2018), esophageal squamous cell carcinoma (Xu et al., 2019), and prostate cancer (Luo et al., 2021). Tian et al. (2018) found that the incidence of microvascular infiltration in patients with *CDCA5* overexpression (45.90%) was higher than that in patients with low *CDCA5* expression (21.92%) in liver cancer. Both *in vivo* and *in vitro* experiments suggested that *CDCA5* inhibition led to a reduced cell-proliferation rate, advocating that *CDCA5* is involved in the malignant progression of HCC.

PDZ-binding kinase (*PBK*) is a serine/threonine kinase belonging to the mitogen-activated protein kinase (*MAPK*) kinase (*MAPKK*) family (Sun et al., 2016). Previous studies have shown that *PBK* is highly trans-activated in various cancers including ovarian cancer (Ma et al., 2022), lymphoma (Wang et al., 2022), kidney cancer (Sun et al., 2022), and colon cancer (Koshino et al., 2022), which is a promising molecular target for cancer-targeted therapy. It is worth mentioning that Yang et al. (2019) found that *PBK* was overexpressed in HCC and was associated with poor prognosis. *PBK* up-regulated *uPAR* expression by enhancing the binding of *ETV4* to the *uPAR* promoter, thereby promoting the invasion and migration of HCC cells. Cao et al. (2021) further explored the role of *PBK* in oxaliplatin resistance of HCC cells, and indicated that *PBK* was further increased in oxaliplatin-resistant HCC cells. Silencing of *PBK* promoted the sensitivity of drug-resistant HCC cells to oxaliplatin, whereas an overexpression of *PBK* inhibited OXA-induced apoptosis. However, due to the lack of relevant studies, the role of *PBK* in liver cancer cannot be fully summarized, and its specific mechanism remains unclear. Through GSEA analysis, we found that *PBK* may be related to tumor angiogenesis, especially the *VEGFR* pathway and *PDGFR* pathway, which rank high among the enrichment pathways of *PBK*-related genes. We verified the effect of *PBK* expression in hepatocellular carcinoma on the proliferation and migration of HCC cells. In addition, we used a tube formation assay to further verify whether *PBK* may promote microtubule formation compared with the control cells. In view of the significant contribution of tumor vasculogenesis in the proliferation of malignant tumors, further exploration of the role of *PBK* in promoting tumor angiogenesis and clarifying its mechanism will provide help for the discovery of therapeutic targets for HCC.

Based on this consequence, down-regulated hub genes were further studied as tumor-suppressor genes. Three genes, *F9*, *C8B*, and *AFM*, were found to be associated with OS, DSS, and PFI. In the correlation analysis with clinicopathological features, *F9* was significantly correlated with T stage, TNM stage, histological grade, vascular invasion, tumor status, AFP, and PT. Furthermore, *F9* has the paramount sensitivity (AUC = 0.882) among the aforementioned three indicators for HCC, suggesting that *F9* is a critical indicator in the diagnosis and prognosis of HCC.

*F9* has been widely studied as a hemophilia defect factor (Lu et al., 2019; Shapiro et al., 2021). Recent research has suggested that *F9* may play a role in *CD4* ^*+*^ T cell induction (Cooper et al., 2009) and predict *CDK4/6* inhibitor response in breast cancer (Carpintero-Fernández et al., 2022). However, the role of *F9* in the immunotherapy of HCC has not been reported. Our study found that *F9* was negatively correlated with the degree of immune infiltration of *CD4* ^*+*^ T cells, neutrophils, B cells, dendritic cells, macrophages, and *CD8* ^*+*^ T cells. The expressions of immune checkpoint-related genes *PD1*, *LAG3*, and *CTLA4* were higher in the group with low *F9* expression. Recent studies have revealed two different mechanisms of tumor immune evasion, including tumor-infiltrating cytotoxic T lymphocyte (CTL) dysfunction and rejection of CTL by immunosuppressive factors. Therefore, the TIDE score can integrate these two mechanisms of tumor immune escape better than widely used biomarkers (tumor mutation load, *PD-L1* levels, and interferon γ) for evaluating the efficacy of immune checkpoint suppressive therapies. In addition, a higher TIDE prediction score in the low *F9* expression group was associated not only with poorer immune checkpoint inhibition treatment but also with poorer survival in patients treated with anti-*PD1* and anti-*CTLA4*, implying that *F9* may be used as an indicator of immune therapy efficacy for HCC. Considering the significance of *F9* in the diagnosis and prognosis of HCC, it is relevant and necessary to expand the clinical sample to explore the role of *F9* in response to immunotherapy.

## Data Availability

The original contributions presented in the study are included in the article/Supplementary Material; further inquiries can be directed to the corresponding author.

## Supplementary material

SUPPLEMENTARY FIGURE S1
**(A–F)** Association between PBK **(A)**, PRC1 **(B)**, CDCA5 **(C)**, CDC20 **(D)**, TOP2A **(E)**, and NCAPG **(F)** expression and disease specific survival (DSS) were performed with the online Kaplan–Meier survival analysis. **(G–L)** Association between PBK **(G)**, PRC1 **(H)**, CDCA5 **(I)**, CDC20 **(J)**, TOP2A **(K)**, and NCAPG **(L)** expressions and progress-free interval (PFI) were performed with the online Kaplan–Meier survival analysis.

SUPPLEMENTARY FIGURE S2
**(A–C)** Association between F9 **(A)**, C8B **(B)**, and AFM **(C)** expressions and DSS was performed. **(D–F)** The correlations between CDC20 **(D)**, TOP2A **(E)**, NCAPG **(F)**, and PFI. **(G–L)** Correlation between KLKB1, ALDH8A1, and CYP2A6 and prognostic indicators DSS **(G–I)** and PFI **(J–L)**.
