# Supplementary material for: Identification of oncogenes and tumor-suppressor genes with hepatocellular carcinoma: A comprehensive analysis based on TCGA and GEO datasets
Source: Front Genet. 2023 Jan 4;13:934883. doi: 10.3389/fgene.2022.934883 (PMC9845404; doi:10.3389/fgene.2022.934883)
Supplement: Supplementary file 1 [file Presentation1.zip › Table 1.DOCX]

Table S1. Sequences of siRNA

| Gene | Sequences |
| --- | --- |
| siPBK#1 | 5′-GAATATGGCAAGAGGGTTAAA-3′ |
| siPBK#2 | 5′-GGGAACTAGGCCACCTATTAA-3′ |
| siPBK#3 | 5′-GAAGTGTGGCTTGCGTAAATA-3′ |
| Negative control | 5′-UUCUCCGAACGUGUCACGUTT-3′ |
