# Supplementary material for: Identification of oncogenes and tumor-suppressor genes with hepatocellular carcinoma: A comprehensive analysis based on TCGA and GEO datasets
Source: Front Genet. 2023 Jan 4;13:934883. doi: 10.3389/fgene.2022.934883 (PMC9845404; doi:10.3389/fgene.2022.934883)
Supplement: Supplementary file 1 [file Presentation1.zip › Table 2.DOCX]

Table S2. Differences between the study and the previous studies

| Study | Gene Source | Design | Methods | Results |
| --- | --- | --- | --- | --- |
| Shen et al. [5] | TCGA database and GSE14520 | Differential analysis was employed to obtain the DEGs. The lasso regression analysis was applied to build the prognosis model. | [Analysis by bioinformatics methods](https://dict.youdao.com/w/eng/analysis_by_bioinformatics_methods/#keyfrom=dict.phrase.wordgroup). | These ten immune-related genes (*BIRC5*, *CDK4*, *DCK*, *HSPA4*, *HSP90AA1*, *PSMD2*, *IL1RN*, *PGF*, *SPP1*, and*STC2*) serve as novel targets for antitumor immunity. |
| Gao et al. [6] | TCGA database and GSE25097 | Screen the hub genes. Kaplan-Meier analysis and Cox proportional hazards model screened genes associated with prognosis. | [Analysis by bioinformatics methods](https://dict.youdao.com/w/eng/analysis_by_bioinformatics_methods/#keyfrom=dict.phrase.wordgroup). | *SPP1, AURKA, NUSAP1, TOP2A, UBE2C, AFP, GMNN, PTTG1, RRM2, SPARCL1, CXCL12, FOS, DCN, SOCS3, FOSB* and *PCK1* can be used as diagnostic biomarkers for liver cancer, among which *FOBS* and *SPP1* genes can also be used as prognostic biomarkers. |
| Yan et al. [7] | TCGA database and GEO datasets | Identified DEGs and established a prognostic model. Investigated the role of signature genes in the HCC microenvironment. | [Analysis by bioinformatics methods](https://dict.youdao.com/w/eng/analysis_by_bioinformatics_methods/#keyfrom=dict.phrase.wordgroup). | Establish a six-gene prognostic signature (*PZP, HMMR, LCAT, GRAMD1C, LPL,* and *ANGPTL1*). These genes may serve as biomarkers in HCC and support personalized disease management. |
| Gao et al. [8] | TCGA database and GEO datasets | Screen DEGs and hub genes, established a prognostic model using univariate Cox proportional hazards regression and LASSO regression. | [Analysis by bioinformatics methods](https://dict.youdao.com/w/eng/analysis_by_bioinformatics_methods/#keyfrom=dict.phrase.wordgroup). | Eight genes were identified as prognostic genes, and four genes (*FLVCR1, HMMR, NEB,* and *UBE2S*) were detrimental genes. All prognostic genes had a strong positive correlation with immune infiltration. |
| Yang et al. [9] | TCGA and CCLE databases | Explore the expression of *TCERG1* in HCC. Analysis the effects of *TCERG1* on liver cancer biological function and immune infiltrates. | Bioinformatics analysis combined with cell function assay. | *TCERG1* is highly expressed in HCC, which is associated with the poor prognosis of liver cancer. |
| Ding et al. [10] | TCGA database and GEO datasets | The differentially expressed genes were identified and the oncogene *CFHR4* was targeted. IHC, qRT-PCR and WB were used for verification. | Bioinformatics analysis combined with clinical tissue specimen validation. | *CFHR4* expression was low in HCC and was significantly related to the poor prognosis of HCC and the level of immune infiltration. |
| Shen et al. [11] | TCGA database | Investigated correlations of various molecular features with antitumor immune signatures and an immunosuppressive signature in HCC. | [Analysis by bioinformatics methods](https://dict.youdao.com/w/eng/analysis_by_bioinformatics_methods/#keyfrom=dict.phrase.wordgroup). | The mutations of several genes significantly correlated with reduced antitumor immune signatures, including *TTN, CTNNB1, RB1, ZFHX4*, and *TP53*. *Syk, Lck, STAT5,* and *Caspase-7* had significant positive expression correlations with *CD8* T cell enrichment and *PD-L1* expression in HCC. |
| Long et al. [12] | TCGA and ImmPort databases | Differentially expressed IRGs were identified. ESTIMATE and CIBERSORT were used to estimate immune cell infiltration in the TIME. | [Analysis by bioinformatics methods](https://dict.youdao.com/w/eng/analysis_by_bioinformatics_methods/#keyfrom=dict.phrase.wordgroup). | Five IRGs were selected by the LASSO Cox model, including *SPP1*, *BIRC5, STC2, GLP1R* and *RAET1E*. The 5-IRG signature may play an important role in mediating immune escape and immune resistance in the TIME of HCC. |
| Chen et al. [13] | TCGA database and GEO datasets | Constructing an IRG signature for HCC and validating its prognostic value in clinical application. | [Analysis by bioinformatics methods](https://dict.youdao.com/w/eng/analysis_by_bioinformatics_methods/#keyfrom=dict.phrase.wordgroup). | Construct an IRG signature comprising nine IRGPs, which were associated with tumor immune mechanisms and prognosis. |
| Our study | TCGA database and GEO datasets | The DEGs were identified, the hub oncogenes and tumor suppressor genes were screened, and their expression and function were explored combined with cell experiments. | Bioinformatics analysis combined with cell function assay. | *CDCA5, CDC20, PBK, PRC1, TOP2A* and *NCAPG* are good indicators of HCC diagnosis and prognosis. Low expression of *F9, AFM,* and *C8B* indicates malignant progression of HCC. *PBK* may promote tumor proliferation through angiogenesis, and *F9* may be a predictor of tumor immunotherapy response. |
