# Supplementary figures and images for: Identification of oncogenes and tumor-suppressor genes with hepatocellular carcinoma: A comprehensive analysis based on TCGA and GEO datasets
Source: Front Genet. 2023 Jan 4;13:934883. doi: 10.3389/fgene.2022.934883 (PMC9845404; doi:10.3389/fgene.2022.934883)

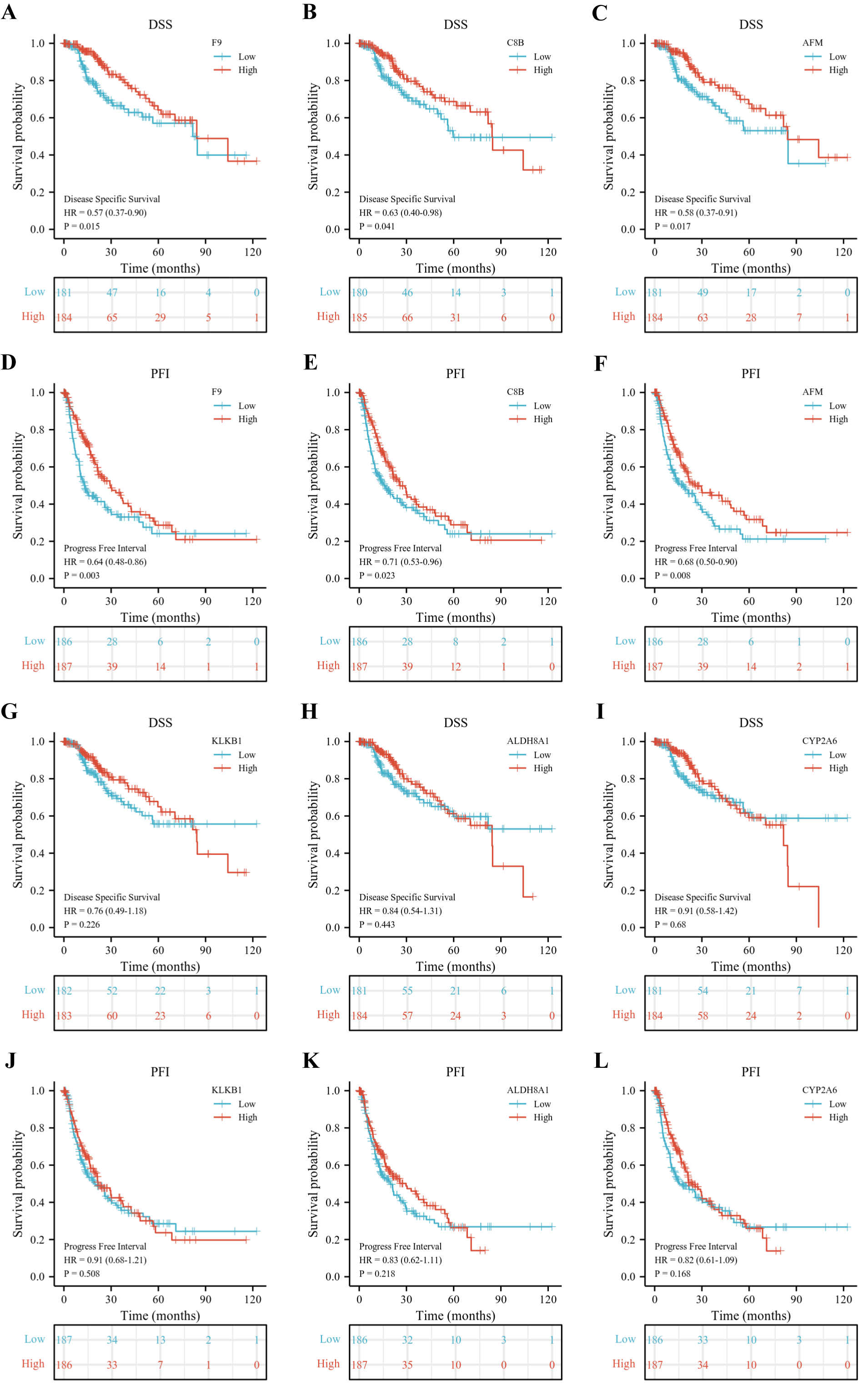

Supplement: Supplementary file 2 [file Image2.TIF]

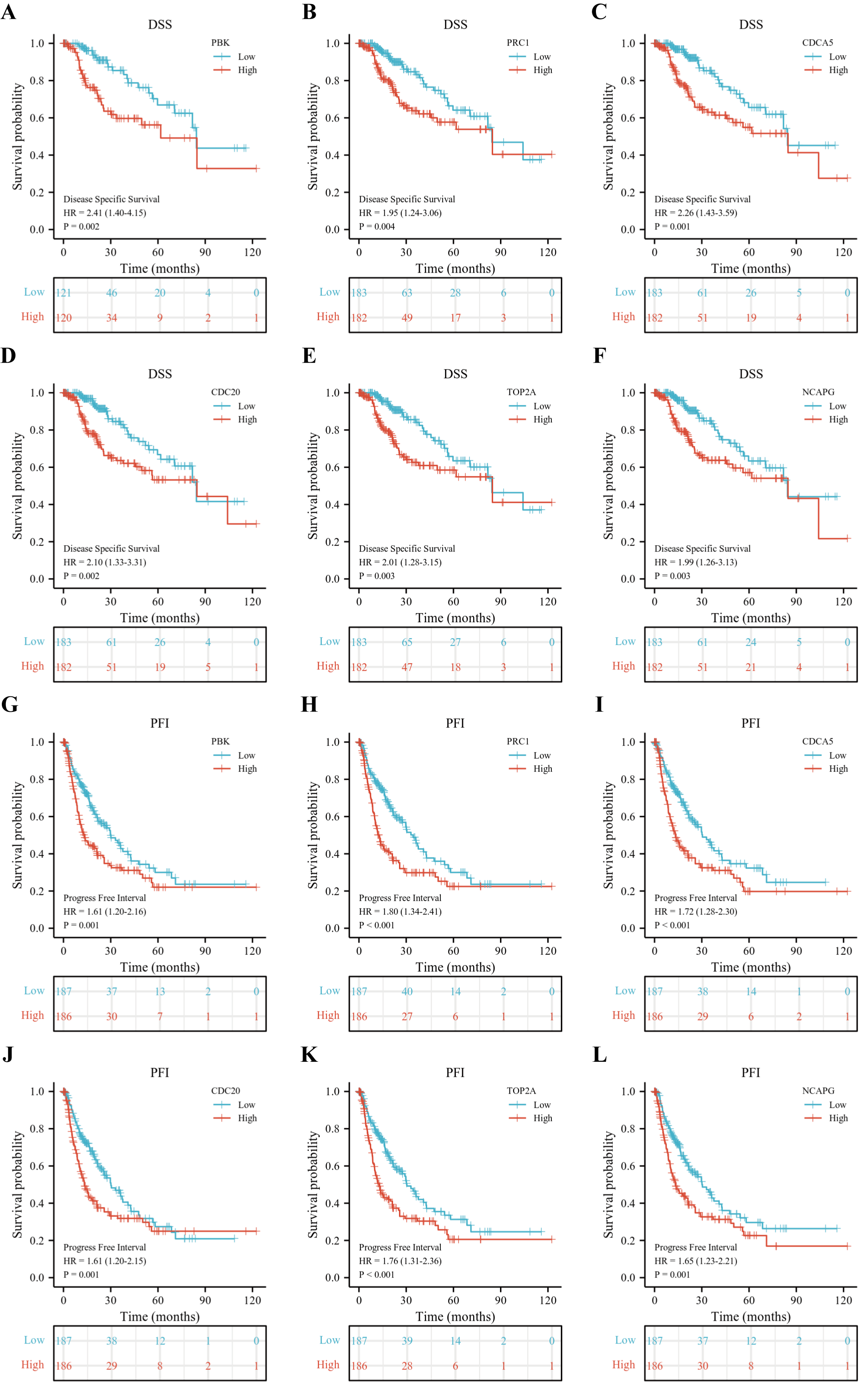

Supplement: Supplementary file 3 [file Image1.TIF]
